# Synergistic effects of genetic susceptibility and air pollution on cardiovascular disease

**DOI:** 10.1016/j.ajpc.2025.101381

**Published:** 2025-12-11

**Authors:** Yun-Jiu Cheng, Li-Juan Liu, Su-Hua Wu, Li-Chun Wang, Hai Deng, Hui-Qiang Wei, Wei-Dong Lin, Xian-Hong Fang, Yi-Jian Liao, Shu-Lin Wu, Yu-Mei Xue, Yue-Dong Ma, Yang Wu

**Affiliations:** a Department of Guangdong Cardiovascular Institute, Guangdong Provincial People’s Hospital, Guangdong Academy of Medical Sciences, Southern Medical University, Guangzhou, China; b Department of Cardiology, the First Affiliated Hospital, Sun Yat-Sen University, Guangzhou, China; c NHC Key Laboratory of Assisted Circulation, Sun Yat-Sen University, Guangzhou, China; d The First Clinical Medical College, Guangdong Medical University, Zhanjiang, China

**Keywords:** Air pollution, Genetic susceptibility, Cardiovascular disease, Polygenic risk score

## Abstract

**Background:**

Although both genetic susceptibility and air pollution are established risk factors for cardiovascular disease (CVD), evidence for their interaction, particularly on the additive scale, remains limited and inconclusive. We aimed to investigate the individual and joint effects of long-term exposure to air pollutants and polygenic risk on incident CVD.

**Methods:**

In a prospective cohort of 460,572 participants from the UK Biobank, we estimated hazard ratios (HRs) for CVD associated with particulate matter (PM_2.5_ and PM_10_), nitrogen dioxide (NO_2_), and nitrogen oxides (NO*_X_*) using Cox models. A polygenic risk score (PRS) for CVD was constructed, and additive and multiplicative interactions between PRS and air pollution were assessed.

**Results:**

Over a median follow-up of 11.92 years, 48,690 incident CVD cases occurred. Both a higher genetic risk and increased air pollution exposure were independently associated with elevated CVD risk. Notably, a significant synergistic effect was observed. Compared to participants with low genetic risk and low pollution exposure, those with high genetic risk and high exposure faced the greatest hazard, with HRs of 1.76 (1.68–1.84) for PM_2.5_, 1.74 (1.66–1.83) for PM_10_, 1.82 (1.73–1.91) for NO_2_, and 1.81 (1.73–1.90) for NO*_x_*. These associations persisted at concentrations below WHO air quality guidelines and were robust across a series of sensitivity analyses.

**Conclusions:**

Our findings call for a reevaluation of air quality standards and indicate that genetic profiling could identify subpopulations that would derive the greatest benefit from air pollution mitigation strategies.

**Figure:**
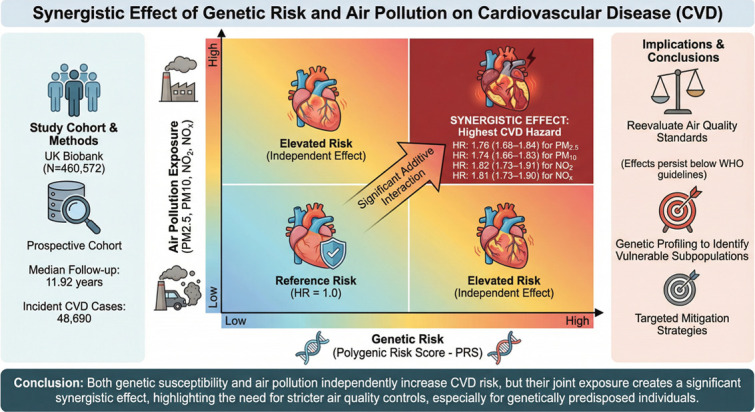
Central illustration.

## 1 Introduction

Cardiovascular disease (CVD) remains a global public health concern, accounting for a substantial proportion of morbidity and mortality worldwide [1,2]. Despite advancements in prevention and treatment strategies, the increasing prevalence of CVD underscores the urgency to identify and address modifiable risk factors. A crucial aspect of this endeavor lies in understanding the intricate interplay between environmental exposures and genetic predisposition.

Air pollution represents a critical environmental threat to cardiovascular health. Particulate matter (PM_2.5_, PM_10_) and nitrogen oxides (NO_2_, NO*_x_*) have been implicated in the development and exacerbation of CVD [[3], [4], [5], [6]]. While both genetic susceptibility and air pollution are established independent risk factors, the extent to which they interact to modify CVD risk remains incompletely defined [[7], [8], [9]]. A recent analysis by Rhee et al. within the UK Biobank reported no significant interaction between PM_2.5_ and genetic risk, highlighting the ongoing uncertainty [10].

Building upon this foundation, our study leverages the full scale and depth of the UK Biobank to address key unresolved questions. We hypothesize that genetic susceptibility not only contributes to CVD risk independently but also acts synergistically with air pollution, and that these effects persist even at pollutant concentrations below current World Health Organization (WHO) air quality guidelines. To test this, we: (1) investigate a comprehensive panel of air pollutants (PM_2.5_, PM_10_, NO_2_, and NO*_x_*); (2) formally test for interaction on both multiplicative and additive scales; and (3) rigorously examine risk relationships at low exposure levels. Our aim is to provide robust evidence to inform both public health strategies and personalized preventive approaches.

## 2 Methods

### 2.1 Study design and participant cohort

The UK Biobank, initiated in 2006, has achieved a remarkable feat by enrolling over half a million community-residing participants aged between 37 and 73 years. These participants were recruited from 22 centers across the United Kingdom [11]. The UK Biobank has amassed a vast array of biological and medical data through the utilization of touch-screen questionnaires, face-to-face interviews, the acquisition of health records, physical examinations, biological sample collections, and imaging procedures. The data repository of the UK Biobank is openly accessible to approved researchers, providing a unique resource for biomedical research.

### 2.2 Assessment of outcome

In our study, incident CVD events were defined as the first occurrence of non-fatal coronary artery disease (CAD, including myocardial infarction, angina pectoris, and coronary revascularization procedures), heart failure (I50), or cerebrovascular disease (including ischemic and hemorrhagic stroke, I60-I64), as captured from hospital inpatient records. Coronary revascularization was identified using OPCS-4 procedure codes K40 through K50. Deaths with a primary cause attributed to any cardiovascular disease (ICD-10 I00-I99) were also included. Importantly, we excluded any UK Biobank participants with a pre-existing history of CVD events that occurred prior to the start of our study period by screening their medical histories and records. Notably, incident events occurring after the completion of the accelerometer measurements, which were conducted to assess physical activity levels and their potential relationship to CVD risk, were deemed as incident cases. The follow-up period for participants encompassed the occurrence of an incident CVD, death, withdrawal from the study, or the respective study endpoints of 30 September 2021 for England, 31 July 2021 for Scotland, and 31 March 2016 for Wales, whichever came first.

### 2.3 Air pollution estimates

Utilizing the follow-up period of the UK Biobank, we acquired annual average concentration data of air pollutants from the Department for Environment, Food and Rural Affairs (DEFRA). DEFRA is renowned for collecting and disseminating high-resolution near-surface air pollution data across the UK (https://uk-air.defra.gov.uk), which has been extensively utilized in published studies [6,12,13]. Through an air dispersion model, DEFRA produces annual concentration maps of air pollutants at a 1 × 1 km resolution. This model incorporates data from the National Atmospheric Emissions Inventory, measurements of secondary inorganic aerosols, and modeling of sources, including dust resuspension. The estimated concentrations are further calibrated against measured concentrations gathered from background sites within DEFRA's Automatic Urban and Rural Network. To ensure the robustness of the models, DEFRA conducts a series of comparisons between the modeled and measured annual mean air pollutants, demonstrating a strong agreement between the two. Summary statistics pertaining to the model's performance are publicly available at https://uk-air.defra.gov.uk/data/pcm-data. To estimate individual exposure, we linked each participant’s residential address history (provided as annual records in UK Biobank) to the corresponding annual pollution grid for each calendar year of follow-up. This method accounts for both spatial variation (using the 1×1 km grid) and temporal variation in pollutant concentrations across the study period. Annual average exposures were updated for each participant year-by-year, thereby creating a time-varying covariate in the Cox models.

Drawing from previous research, we estimated the exposure levels of each participant to PM_2.5_, PM_10_, NO_2_, and NO*_x_* [14]. Leveraging the participants' residential address history obtained from the UK Biobank, we assigned the annual average air pollutant concentrations to each participant based on a unique code (ukgridcode) that corresponds to each 1 × 1 km cell in the annual concentration map. This approach allows for a precise estimation of air pollutant exposure for each individual, enabling us to further investigate its potential impact on health outcomes.

### 2.4 Genetic data and polygenic risk score calculation

We evaluated the genetic predisposition to CVD utilizing two distinct approaches. Initially, we employed the polygenic risk score (PRS) as an indicator of an individual's genetic vulnerability to CVD. Specifically, following rigorous quality control measures for genome-wide association studies (GWAS), we incorporated genotyping data from 376,833 participants in the computation of PRS [15]. This PRS was derived using penalised regression (LASSO) implemented in the bigsnpr R package, based on the summary statistics of a GWAS on coronary artery disease conducted by the CARDIoGRAMplusC4D Consortium, which encompassed 60,801 cases and 123,504 controls from 48 studies across eight countries [16]. This method automatically selects informative single nucleotide polymorphisms (SNPs) and estimates their weights while accounting for linkage disequilibrium, using the external GWAS summary statistics as a prior. The resulting score represents an individual's genetic liability for CAD, a core component of our composite CVD endpoint. In our data, this PRS was strongly associated with incident CVD risk per standard deviation increase (odds ratio 1.21, 95 % CI 1.21–1.23). Participants were then categorized into low (Tertile 1), intermediate (Tertile 2), and high (Tertile 3) genetic risk groups based on this PRS. Detailed parameters are provided in Supplementary Text S1.

During the validation phase, the PRS for CVD exhibited a robust association with CVD phenotypes in our dataset. This association was quantified using a logistic regression model, adjusting for birth year, sex, genotyping batch, and the first ten principal components to account for population heterogeneity. The odds ratios obtained were 1.21, with a 95 % confidence interval (CI) ranging from 1.21 to 1.23 for every unit increase in PRS. Subsequently, the study participants were stratified into three distinct genetic risk categories: low (Tertile 1), intermediate (Tertile 2), and high (Tertile 3), based on their PRS.

### 2.5 Ascertainment of covariates

In our study, we considered several potential confounders, including age, sex, ethnicity, educational attainment, alcohol consumption status, tobacco consumption status, healthy diet score, body mass index (BMI), physical activity, blood pressure levels, and prevalent diseases. Specifically, physical activity was assessed using the Metabolic Equivalent Task (MET) minutes derived from the abbreviated International Physical Activity Questionnaire (IPAQ). The healthy diet score was determined based on the following dietary factors: consumption of vegetables ≥ four servings per day, fruits ≥ three pieces per day, fish ≥ twice per week, unprocessed red meat ≤ twice per week, and processed meat ≤ twice per week. Each favorable dietary factor contributed one point, resulting in a healthy diet score ranging from 0 to 5. During the baseline assessment visit, height and weight were measured by trained nurses, and BMI was calculated as the ratio of weight in kilograms to the square of height in meters. The participants' self-reported information and medical records were used to determine their history of hyperlipidemia, hypertension, and diabetes. Systolic blood pressure (SBP) and diastolic blood pressure (DBP) were measured at baseline using standardized protocols administered by trained nurses, and the mean values of two automated or manual measurements were recorded.

### 2.6 Statistical analysis

Descriptive statistics were utilized to characterize the study variables. Specifically, for continuous variables that followed a normal distribution, we reported the mean, standard deviation (SD), and additionally provided the 5th/95th percentiles to offer a more comprehensive view of the data distribution. Conversely, for those continuous variables that exhibited skewed distributions, we presented the median and range. For categorical data, we provided percentages to represent the distribution of values. To assess differences in characteristics among distinct groups in our study, we employed appropriate statistical methodologies tailored to the nature of the data. We adopted χ² tests for categorical variables, the Wilcoxon–Mann–Whitney U test for non-parametric continuous data exhibiting skewed distributions, and Student's *t*-test for parametric continuous data that followed a normal distribution.

To quantify the associations between long-term exposure to air pollutants and the risk of incident CVD, we implemented the Cox proportional hazards model with time-varying measurements of exposure. To ensure robust estimates, we adjusted for several potential confounders, including UK Biobank assessment center, age, sex, ethnicity, educational attainment, alcohol consumption status, tobacco consumption status, healthy diet score, physical activity level, BMI, SBP, DBP, and the presence of hyperlipidemia, hypertension, and diabetes. The results were presented as hazard ratios (HRs) with their corresponding 95 % confidence intervals (CIs), which provide insights into the magnitude and direction of the associations between air pollutant exposure and CVD risk.

To investigate the dose-response associations between air pollutants and the risk of incident CVD, a restricted cubic spline model incorporating four knots was employed. Furthermore, to ascertain whether these relationships persist even at lower levels of exposure, specific analyses were undertaken among individuals exposed to air pollutant concentrations below the World Health Organization (WHO) air quality guideline limits applicable at the time of this analysis. For direct comparison with a large body of existing cohort studies and to maintain consistency with the exposure distribution in our cohort, we employed the WHO 2005 global Air Quality Guidelines (AQG) interim targets as our primary reference thresholds [17]: specifically, annual mean concentrations of PM_2.5_ <10 μg/m^3^, PM_10_ <20 μg/m^3^, and NO_2_ <40 μg/m^3^. Analyses for NO_x_ were conducted using a comparable threshold of <40 μg/m^3^. This approach allows for a meaningful assessment of risk at levels commonly deemed ‘safe’ in recent epidemiological contexts and facilitates comparison with prior literature [17,18].

To evaluate the potential modification of genetic factors on the relationships between air pollutants and CVD risk in a multiplicative framework, we incorporated an interaction term into our statistical model to derive the *P* for interaction. On an additive scale, we utilized the Bootstrap method to estimate the relative excess risk due to interaction (RERI), the attributable proportion (AP) due to interaction, and the Synergy Index (SI), along with their respective 95 % CIs. On an additive scale, we utilized the Bootstrap method to estimate the relative excess risk due to interaction (RERI), the attributable proportion (AP) due to interaction, and the Synergy Index (SI), along with their respective 95 % CIs. RERI = HR11 – HR10 – HR01 + 1, where HR11 is the hazard ratio for joint exposure, and HR10 and HR01 are the hazard ratios for each exposure alone. AP = RERI / HR11, representing the proportion of risk in the doubly exposed group attributable to the interaction. SI = (HR11 – 1) / [(HR10 – 1) + (HR01 – 1)]. If the CIs of RERI and AP exclude zero, or the CIs of SI excludes one, it indicates the presence of an additive interaction. If the CIs of RERI and AP exclude zero, or the CIs of SI excludes one, it indicates the likelihood of an additive interaction [19]. This approach enabled us to comprehensively assess the interaction between genetic factors, air pollution, and CVD risk.

To test the robustness of our findings, we conducted a series of sensitivity analyses: (1) Excluding participants diagnosed with CVD within the first three years of follow-up to minimize reverse causality; (2) Accounting for competing risk of non-cardiovascular death using the Fine and Gray subdistribution hazards model; (3) Performing a complete-case analysis excluding participants with missing covariates; (4) Restricting analyses to participants who self-reported good health at baseline; (5) Restricting to participants residing at their current address for >5 years to reduce exposure misclassification; (6) Fitting two-pollutant models to examine confounding between pollutants; and (7) Conducting stratified analyses by key demographic and clinical factors. Of note, we calculated the population attributable fraction (PAF) to estimate the proportion of CVD cases that could be theoretically prevented if all participants had an exposure level equivalent to the first quartile (Q1), assuming a causal relationship between air pollution and CVD. PAFs were calculated overall and stratified by genetic risk category using the adjusted hazard ratios. (8) Conducting a combined sensitivity analysis adjusting for three additional covariates simultaneously: the Townsend deprivation index (area-level socioeconomic status), proximity to a major road (≤200 m vs. >200 m), and area-level urban-rural classification. Statistical tests were two-sided, and p-values < 0.05 were considered statistically significant. All analyses were conducted in Stata version 16.0 (StataCorp LP, College Station, TX) and R version 4.2.2 (R Foundation for Statistical Computing).

## 3 Results

### 3.1 Study population characteristics

The baseline demographic and clinical characteristics of the 460,572 participants are presented in Table 1, stratified by the occurrence of incident CVD. The mean age of the cohort was 56.10 (±8.09) years. Over the median follow-up period of 11.92 years, we identified 48,690 incident CVD cases. Participants who experienced incident CVD were significantly older and predominantly male, compared to those who remained free of CVD. Additionally, those with incident CVD exhibited lower educational attainment and a higher BMI compared with those without incident CVD. Participants with incident CVD exhibited a higher likelihood of being former or current smokers compared to those without incident CVD. Current alcohol consumption and adherence to a healthy diet were less prevalent among those with incident CVD. Participants with incident CVD were more likely to engage in physical activity. Furthermore, a higher prevalence of hyperlipidemia, hypertension, and diabetes mellitus, as well as elevated baseline blood pressure levels, was observed among participants who developed incident CVD. The mean (SD) estimates of PM_2.5_, PM_10_, NO_2_, and NO*_x_* were 10.02 (1.06), 19.23 (1.89), 29.16 (8.76), and 44.49 (15.55) μg/m³, respectively, among participants with incident CVD; and the corresponding concentrations were 9.98 (1.05), 19.22 (1.96), 29.04 (9.13), 43.96 (15.47) μg/m^3^ for those without incident CVD.

**Table 1:** Baseline characteristics of the participants included in the study.

Baseline characteristics	Total participants (N = 460,572)		Participants without CVD (N = 411,882)	Participants with CVD (N = 48,690)	P-values
Age (years)		56.10 (8.09)	55.62 (8.08)	60.16 (6.98)	<0.001
Female sex, n ( %)		258,694 (56.17)	238,602 (57.93)	20,092 (41.27)	<0.001
Ethnicity, n ( %)					0.46
White Europeans		435,076 (94.46)	389,046 (94.46)	46,030 (94.54)	
Non-White Europeans		25,496 (5.54)	22,836 (5.54)	2660 (5.46)	
Education, n ( %)					<0.001
Degree-level education		152,372 (33.08)	139,945 (33.98)	12,427 (25.52)	
Non-college education		308,200 (66.92)	271,937 (66.02)	36,263 (74.48)	
BMI (kg/m^2^)		27.28 (4.74)	27.13 (4.67)	28.55 (5.14)	<0.001
Tobacco consumption status, n ( %)					<0.001
Never smokers		259,368 (56.31)	236,426 (57.40)	22,942 (47.12)	
Former smokers		153,958 (33.43)	135,185 (32.82)	18,773 (38.56)	
Current smokers		47,246 (10.26)	40,271 (9.78)	6975 (14.33)	
Alcohol consumption status, n ( %)					<0.001
Never drinkers		21,308 (4.63)	18,685 (4.54)	2623 (5.39)	
Former drinkers		15,347 (3.33)	13,056 (3.17)	2291 (4.71)	
Current drinkers		423,917 (92.04)	380,141 (92.29)	43,776 (89.91)	
Healthy diet score, n ( %)					<0.001
0		8000 (1.74)	6930 (1.68)	1070 (2.20)	
1		42,330 (9.19)	37,306 (9.06)	5024 (10.32)	
2		99,315 (21.56)	88,038 (21.37)	11,277 (23.16)	
3		135,143 (29.34)	120,847 (29.34)	14,296 (29.36)	
4		119,584 (25.96)	107,723 (26.15)	11,861 (24.36)	
5		56,200 (12.20)	51,038 (12.39)	5162 (10.60)	
Physical activity (MET-min/week)		2655.83 (2648.85)	2648.92 (2632.25)	2716.94 (2790.69)	<0.001
Hyperlipidemia ( %)		47,484 (10.31)	39,099 (9.49)	8385 (17.22)	<0.001
Hypertension ( %)		112,510 (24.43)	92,973 (22.57)	19,537 (40.13)	<0.001
Diabetes ( %)		19,887 (4.32)	15,242 (3.70)	4645 (9.54)	<0.001
Systolic blood pressure (mmHg)		135.43 (21.93)	134.68 (21.72)	141.75 (22.62)	<0.001
Diastolic blood pressure (mmHg)		81.03 (12.28)	80.83 (12.22)	82.72 (12.62)	<0.001
PM_2.5_ (μg/m^3^)		9.98 (1.05)	9.98 (1.05)	10.02 (1.06)	<0.001
PM_10_ (μg/m^3^)		19.22 (1.95)	19.22 (1.96)	19.23 (1.89)	0.51
NO_2_ (μg/m^3^)		29.05 (9.09)	29.04 (9.13)	29.16 (8.76)	0.004
NO_x_ (μg/m^3^)		44.01 (15.48)	43.96 (15.47)	44.49 (15.55)	<0.001

Abbreviations: CVD, cardiovascular disease; BMI, body mass index; physical activity (MET-min/week), MET, Metabolic Equivalent Task; PM_2.5_, particular matter with aerodynamic diameter ≤ 2.5 mm; PM_10_, particular matter with an aerodynamic diameter ≤ 10 mm; NO_2_, nitrogen dioxide; NO_x_, nitrogen oxides.

### 3.2 Air pollution and CVD

**Fig. 1:**
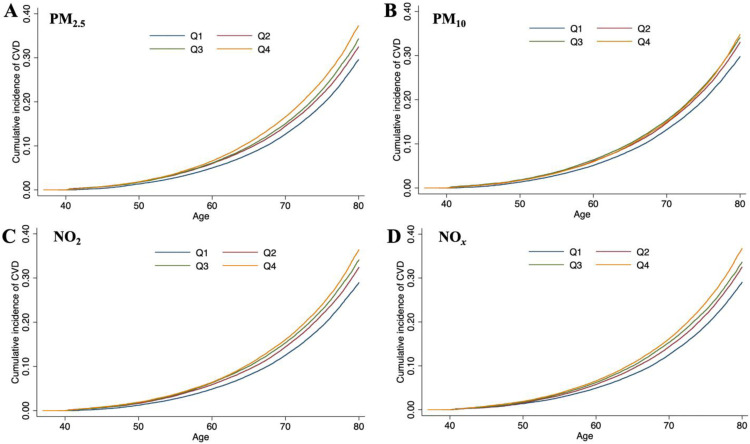
Associations of exposure to PM_2.5_ (A), PM_10_ (B), NO_2_ (C), and NO_x_ (D) with the risk of incident cardiovascular disease incidence, analyzed using the Fine–Grey proportional sub-distribution hazards model.

**Fig. 2:**
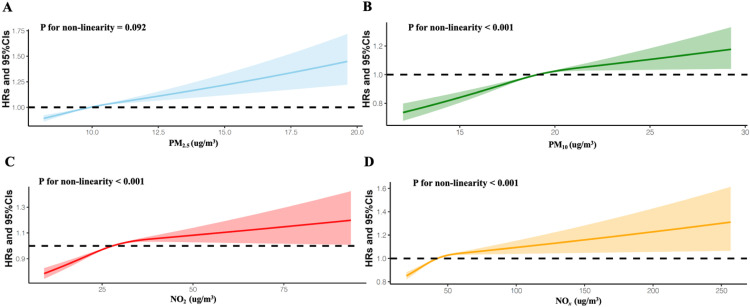
Associations of PM_2.5_ (A), PM_10_ (B), NO_2_ (C), and NO_x_ (D) exposure with the risk of cardiovascular disease events among participants in the UK Biobank.

Exposure to air pollutants over a long-term period substantially elevates the risk of CVD, as delineated in Table 2. The HRs (95 % CIs) were as follows: 1.05 (1.04–1.06) for PM_2.5_ per 1.06 μg/m^3^ increment, 1.05 (1.04–1.07) for PM_10_ per 1.92 μg/m^3^ increment, 1.06 (1.05–1.08) for NO_2_ per 8.77 μg/m^3^ increment, and 1.04 (1.03–1.05) for NO*_x_* per 15.50 μg/m^3^ increment. The analysis of the data, when stratified by air pollutant exposure levels, yielded a statistically significant trend (*P* < 0.001). Individuals with higher exposure to air pollutants demonstrated a markedly elevated cumulative incidence of CVD relative to those with lower exposure levels, as illustrated in Fig. 1. Upon confining the analysis to individuals with minimal exposure to air pollutants, the adverse outcomes associated with air pollutants exposure were observed to persist. Even at low levels of exposure, there was an increase in the risk of incident CVD attributable to exposure to PM_2.5_, PM_10_, NO_2_, and NO*_x_* (see Supplementary Table S2). Specifically, the relative risk increments for PM_2.5_, PM_10_, NO_2_, and NO*_x_* exposure were approximately 2 %, 3 %, 5 %, and 4 %, respectively. This finding underscores the pervasive impact of air pollutants on cardiovascular health, even at exposure levels that are traditionally considered to be low. Fig. 2 displays the dose–response curves of PM_2.5_, PM_10_, NO_2_, and NO*_x_* concentrations with the occurrences of CVD. We found a monotonic increase in the dose–response relationships between PM_2.5_ concentrations and the risk of CVD (*P* for nonlinearity > 0.05). Of note, dose–response curves for PM_10_, NO_2_, and NO*_x_* initially demonstrated a monotonic increase within the lower range of exposure levels, while the dose-response curves showed a slight decrease or plateau when participants were exposed to high levels of air pollutants (*P* for nonlinearity < 0.001).

**Table 2:** Adjusted hazard ratios and 95 % confidence interval for air pollution concentrations with the risk of incident cardiovascular disease in the UK Biobank study.

Air pollutants	HRs (95 % CIs)	P-values	P for trend
PM_2.5_			
PM_2.5_-Q1	Ref.		<0.001
PM_2.5_-Q2	1.05 (1.02, 1.08)	<0.001	
PM_2.5_-Q3	1.07 (1.05, 1.10)	<0.001	
PM_2.5_-Q4	1.14 (1.11, 1.17)	<0.001	
PM_2.5_, per SD^a^ increase	1.05 (1.04, 1.06)	<0.001	
PM_10_			
PM_10_-Q1	Ref.		<0.001
PM_10_-Q2	1.06 (1.03, 1.08)	<0.001	
PM_10_-Q3	1.10 (1.07, 1.13)	<0.001	
PM_10_-Q4	1.13 (1.10, 1.17)	<0.001	
PM_10_, per SD^b^ increase	1.05 (1.04, 1.07)	<0.001	
NO_2_			
NO_2_-Q1	Ref.		<0.001
NO_2_-Q2	1.06 (1.03, 1.09)	<0.001	
NO_2_-Q3	1.10 (1.08, 1.13)	<0.001	
NO_2_-Q4	1.18 (1.15, 1.22)	<0.001	
NO_2_, per SD^c^ increase	1.06 (1.05, 1.08)	<0.001	
NO_x_			
NO_x_-Q1	Ref.		<0.001
NO_x_-Q2	1.07 (1.04, 1.10)	<0.001	
NO_x_-Q3	1.09 (1.06, 1.12)	<0.001	
NO_x_-Q4	1.15 (1.12, 1.19)	<0.001	
NO_x_, per SD^d^ increase	1.04 (1.03, 1.05)	<0.001	

HR, hazard ratio; CI, confidence interval; PM_2.5_, particular matter with aerodynamic diameter ≤ 2.5 mm; PM_10_, particular matter with an aerodynamic diameter ≤ 10 mm; NO_2_, nitrogen dioxide; NO_x_, nitrogen oxides.

1Adjusted for UK Biobank assessment center, age, sex, ethnicity, educational attainment, alcohol consumption status, tobacco consumption status, healthy diet score, physical activity (MET-min/week), body mass index (kg/m^2^), systolic blood pressure (mmHg), and diastolic blood pressure (mmHg), and the presence of hyperlipidemia, hypertension, and diabetes.

a The standard deviation of PM_2.5_ was 1.06 μg/m^3^.

b The standard deviation of PM_10_ was 1.92 μg/m^3^.

c The standard deviation of NO_2_ was 8.77 μg/m^3^.

d The standard deviation of NOx was 15.50 μg/m^3^.

### 3.3 Genetic susceptibility and CVD

Upon stratification of the PRS into low, intermediate, and high genetic risk levels—individuals within the intermediate and high genetic risk categories exhibited a significantly elevated risk of developing CVD. Specifically, those at intermediate genetic risk had a 23 % (20 %, 26 %) increased risk, whereas those at high genetic risk had a 56 % (52 %, 60 %) increased risk, when compared to their counterparts in the low genetic risk group (see Supplementary Table S3). Furthermore, the analysis of the cumulative incidence curves, stratified by genetic risk, corroborated these findings, demonstrating a parallel trend in the risk of CVD across the different genetic risk strata (Supplementary data online, Figure S1).

### 3.4 Joint effects of air pollution and genetic risk

**Fig. 3:**
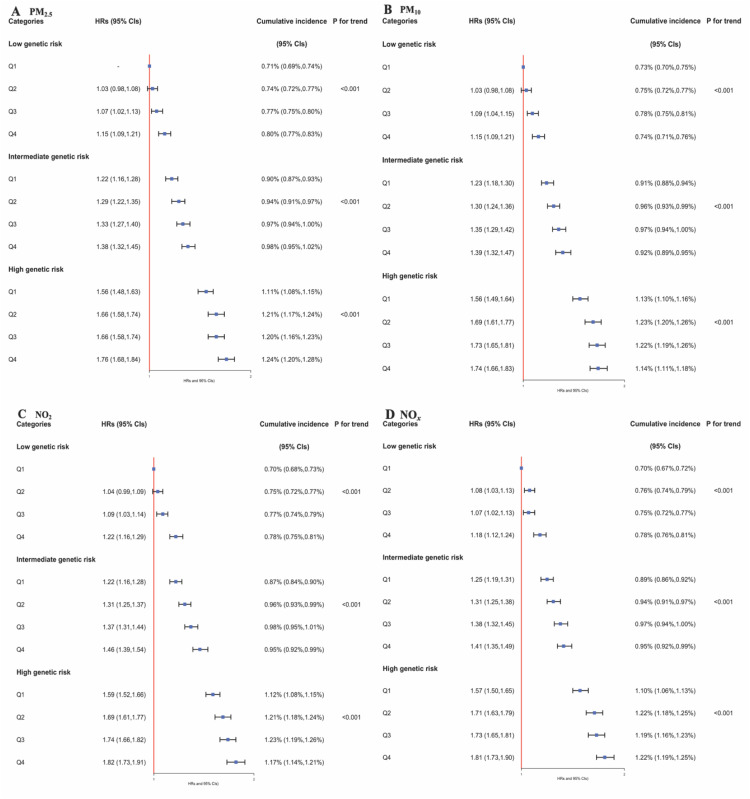
The joint associations of exposure to PM_2.5_ (A), PM_10_ (B), NO_2_ (C), and NO_x_ (D) (categorized into quintiles: Q1-Q4) and the incident cardiovascular disease polygenic risk score (PRS, categorized into tertiles: low, intermediate, and high) with the risk of incident cardiovascular disease, examined using the Cox proportional hazards model.

In an exploration of the combined influence of air pollutants and genetic predisposition on CVD risk, a significant increase in CVD event risk was observed among individuals with elevated exposure to air pollutants and a high genetic risk profile (Fig. 3). Of note, compared to participants with low exposure and genetic risk, those with high exposure and risk had increased risks of CVD associated with PM_2.5_ [1.76 (95 % CI 1.68–1.84)], PM_10_ [1.74 (95 % CI 1.66–1.83)], NO_2_ [1.82 (95 % CI 1.73–1.91)], and NO*_x_* [1.81 (95 % CI 1.73–1.90)]. In Table 3, we examined the relative excess risks due to RERI, AP, and SI for PM_2.5_, PM_10_, NO_2_, and NO*_x_* across varying levels of genetic risk. The results indicated no significant additive interaction for individuals with medium genetic risk, as evidenced by RERI and AP confidence intervals that included the null. However, those in the high genetic risk category exhibited positive RERI values, particularly in the upper quintiles for all pollutants, suggesting additive interactions. Furthermore, an SI greater than one was observed in this group, implying a potential multiplicative effect of the combined influence of genetic susceptibility and air pollutant exposure on CVD risk.

**Table 3:** Additive and multiplicative interactions between air pollutants and PRS on the risk of incident CVD among participants in the UK Biobank.

	RERIs (95 % CIs)	APs (95 % CIs)	SIs (95 % CIs)
PM_2.5_			
Medium genetic risk			
PM_2.5-_Q2	-0.02 (-0.09, 0.01)	-0.01 (-0.04, 0.02)	0.96 (0.89, 1.03)
PM_2.5-_Q3	0.02 (-0.01, 0.03)	0.01 (-0.002, 0.04)	1.06 (0.97, 1.16)
PM_2.5-_Q4	-0.001 (-0.02, 0.01)	-0.001 (-0.01, 0.01)	1.00 (0.96, 1.03)
High genetic risk			
PM_2.5-_Q2	0.02 (-0.01, 0.03)	0.01 (0.001, 0.03)	1.04 (0.99, 1.08)
PM_2.5-_Q3	0.01 (0.001, 0.02)	0.01 (0.003, 0.02)	1.03 (1.00, 1.06)
PM_2.5-_Q4	0.01 (0.002, 0.01)	0.01 (0.003, 0.01)	1.02 (1.01, 1.04)
PM_10_			
Medium genetic risk			
PM_10-_Q2	0.02 (-0.04, 0.05)	0.02 (-0.02, 0.06)	1.10 (0.85, 1.41)
PM_10-_Q3	-0.003 (-0.03, 0.01)	-0.002 (-0.02, 0.02)	0.99 (0.93, 1.06)
PM_10-_Q4	-0.004 (-0.02, 0.01)	-0.003 (-0.01, 0.01)	0.99 (0.95, 1.03)
High genetic risk			
PM_10-_Q2	0.02 (-0.01, 0.03)	0.01 (-0.001, 0.03)	1.06 (0.98, 1.14)
PM_10-_Q3	0.01 (-0.004, 0.01)	0.005 (-0.001, 0.01)	1.02 (0.99, 1.04)
PM_10-_Q4	0.007 (0.0002, 0.01)	0.005 (0.001, 0.01)	1.02 (1.002, 1.04)
NO_2_			
Medium genetic risk			
NO_2-_Q2	-0.01 (-0.07, 0.02)	-0.01 (-0.03, 0.03)	0.98 (0.91, 1.07)
NO_2-_Q3	0.02 (-0.01, 0.04)	0.02 (-0.001, 0.04)	1.07 (0.98, 1.17)
NO_2-_Q4	0.002 (-0.02, 0.01)	0.001 (-0.008, 0.01)	1.00 (0.97, 1.04)
High genetic risk			
NO_2-_Q2	0.02 (-0.002, 0.03)	0.01 (0.003, 0.03)	1.04 (1.00, 1.08)
NO_2-_Q3	0.02 (0.01, 0.03)	0.02 (0.01, 0.02)	1.06 (1.03, 1.10)
NO_2-_Q4	0.01 (0.005, 0.02)	0.008 (0.004, 0.01)	1.03 (1.01, 1.04)
NO_x_			
Medium genetic risk			
NO_x-_Q2	0.01 (-0.06, 0.04)	0.008 (-0.02, 0.05)	1.03 (0.90, 1.17)
NO_x-_Q3	0.004 (-0.03, 0.02)	0.003 (-0.01, 0.02)	1.01 (0.94, 1.08)
NO_x-_Q4	0.01 (-0.01, 0.02)	0.01 (-0.001, 0.02)	1.03 (0.98, 1.09)
High genetic risk			
NO_x-_Q2	0.02 (-0.003, 0.03)	0.01 (0.003, 0.03)	1.05 (1.00, 1.10)
NO_x-_Q3	0.02 (0.01, 0.03)	0.02 (0.01, 0.02)	1.06 (1.03, 1.09)
NO_x-_Q4	0.01 (0.004, 0.01)	0.007 (0.004, 0.01)	1.03 (1.01, 1.04)

P values, RERIs, APs and 95 % CIs in bold represent significance at P < 0.05. If 0 is outside the CIs of RERIs and APs, it means that there is an additive interaction.

Cox regression models adjusted for UK Biobank assessment center, age, sex, ethnicity, educational attainment, alcohol consumption status, tobacco consumption status, healthy diet score, physical activity (MET-min/week), body mass index (kg/m^2^), systolic blood pressure (mmHg), and diastolic blood pressure (mmHg), and the presence of hyperlipidemia, hypertension, diabetes, birth year, genotyping batch, and the first ten principal components to account for population heterogeneity.

Abbreviations: HRs, hazard ratios; CIs, confidence intervals; PM_2.5_, fine particulate matter with diameter <2.5 μm; PM_10_, particulate matter with diameter <10 μm; NO_2_, nitrogen dioxide; NO_x_, nitrogen oxides; CVD, cardiovascular disease; BMI, body mass index; SBP, systolic blood pressure.

### 3.5 Sensitivity analyses

The robustness of the primary findings was confirmed through a series of sensitivity analyses. The positive associations between air pollutants and CVD risk remained consistent after excluding events within the first three years of follow-up (Supplementary Table S4) and when accounting for competing risks from non-cardiovascular death (Supplementary Table S5). Results were materially unchanged in complete-case analyses without imputation and among participants reporting good health at baseline (Supplementary Tables S6, S7). Crucially, the observed associations were strengthened in participants with stable residence (>5 years at the same address), lending further credibility to our exposure assessment (Supplementary Table S8). In two-pollutant models, the hazardous effects of PM_2.5_ and NO_2_ remained robust after mutual adjustment, whereas the association for PM_10_ was attenuated when adjusted for NO*_x_*, indicating potential shared sources or pathways (Supplementary Table S9). Finally, a combined sensitivity analysis that simultaneously adjusted for area-level deprivation, road proximity, and urban-rural classification yielded results nearly identical to the primary model, with all associations remaining statistically significant (Supplementary Table S12).

### 3.6 Stratified analyses

Stratified analyses demonstrated that the deleterious effects of air pollution were broadly consistent across most demographic subgroups, including age and sex. However, we identified several key subpopulations at particularly heightened risk. The associations were significantly more pronounced among individuals of White European ethnicity, those with lower educational attainment, and those with pre-existing hypertension or diabetes. Notably, for certain pollutants, never-smokers also appeared to be more vulnerable (Supplementary Table S10). These findings highlight specific sociodemographic and clinical factors that may confer increased susceptibility to the cardiovascular effects of air pollution.

### 3.7 Population attributable fraction

To quantify the potential public health impact of air pollution, we estimated the PAF (Supplementary Table S11). In the overall population, exposure to air pollutants in the highest quintile (Q4) accounted for a substantial proportion of CVD cases, with PAFs of 6.8 % for PM_2.5_, 7.2 % for PM_10_, 9.5 % for NO_2_, and 7.8 % for NO_x_. Strikingly, the disease burden attributable to air pollution was not uniform across genetic strata. The PAFs were consistently and significantly highest among individuals with high genetic risk. For example, exposure to the highest quartile of PM_2.5_ explained 10.1 % of CVD cases in the high genetic risk group, compared to only 4.5 % in the low genetic risk group.

## 4 Discussion

This large, prospective cohort study yields several key findings. First, we confirm a significant association between long-term exposure to major air pollutants and incident CVD, which persists even at levels deemed safe by WHO guidelines. Moreover, we show that the risk is most pronounced in individuals with high genetic susceptibility. Most importantly, we provide evidence of a significant additive interaction between genetic predisposition and air pollution, pointing to a synergistic biological effect. By resolving a key gap in prior research that focused predominantly on independent risks, our work offers a more comprehensive framework for understanding CVD etiology.

As established risk factors cannot fully explain CVD incidence, air pollution represents a critical and ubiquitous environmental risk factor. Although numerous clinical trials and observational studies have endeavored to explore individual-level strategies to attenuate the detrimental cardiovascular impact of air pollution, their conclusions remain inadequate [5,[20], [21], [22], [23]]. Notably, studies that solely evaluate the impact of a singular lifestyle factor on CVD tend to overlook a comprehensive assessment of the intricate association between air pollution and CVD, particularly the pivotal role of genetic predisposition. Moreover, the interplay between air pollutants and genetic factors in modulating the relationship between air pollution exposure and CVD remains poorly understood. Moreover, the specific concentration thresholds at which long-term exposure exerts detrimental effects remain inadequately defined. In European regions, the concentrations of air pollutants, particularly PM_2.5_, exhibit a propensity towards adherence to the WHO’s air quality guidelines, positioning them comparatively low on a global scale [24]. However, our meticulous investigation underscores a significant finding: the cardiovascular implications of prolonged exposure to these pollutants persist, even when the exposure levels fall below the WHO's prescribed air quality threshold limits.

The credibility of our findings is strengthened by the comprehensive suite of sensitivity analyses. The persistence of significant associations after accounting for competing risks of death confirms that our results are not merely an artifact of differential mortality. The stability of the hazard ratios across multiple analytical approaches—including complete-case analysis, analysis restricted to healthy participants at baseline, and analysis of long-term residents—effectively mitigates concerns regarding missing data, reverse causality, and exposure misclassification. The observed effect modification by socioeconomic and clinical factors suggests that the cardiovascular burden of air pollution is not uniformly distributed, which has important implications for targeted public health interventions.

The observed associations between air pollution and CVD are likely mediated by several potential mechanisms. Firstly, air pollutants can traverse the alveoli directly into the bloodstream, ultimately depositing on blood vessel walls. This can result in endothelial dysfunction, vasoconstriction, and thrombosis [25,26]. Alternatively, air pollutants may indirectly induce oxidative stress and systemic inflammatory responses, thereby contributing to autonomic dysfunction and accelerating CVD progression [27,28]. Furthermore, as exogenous stimuli, inhaled air pollutants can trigger a chronic systemic inflammatory response, activating associated signaling pathways through inflammatory cytokines, ultimately leading to CVD progression [29,30].

It is intriguing to note that the risk of CVD escalates significantly in the range of low exposure concentrations, while the dose-response curves tend to stabilize as exposure concentrations rise. The observed leveling off or decrease in CVD risk at high concentrations of air pollutants can be attributed to multiple reasons. One possible explanation is that individuals who are more vulnerable to air pollution might have developed symptoms and sought medical attention prior to the pollutants peaking. Furthermore, distinct health risks may be associated with various pollutants, prompting individuals to minimize their outdoor exposure or wear protective face masks during periods of severe air pollution.

Despite prior investigations highlighting the role of genetic susceptibility in the predisposition to CVD, the combined influence of genetic factors and air pollution exposure on CVD incidence remains unexplored [31]. Our study fills this gap by demonstrating a monotonic increase in CVD risk with both elevated genetic risk and heightened exposure to air pollutants. Notably, our findings uncover potential interactions between air pollutants and PRS, indicating a synergistic effect. Considering that CVD-related SNPs and air pollution exposure share some similarities in the biological pathways of CVD initiation and progression, the interactions between genetic susceptibility and air pollutants exposure are biologically plausible. For example, SNPs, such as *CDH13, LPL*, and *APOE* are known to be correlated with cardiovascular conditions [9,[32], [33], [34]]. Our discovery holds significant implications for environmental epidemiology, particularly in the context of providing tailored preventive or therapeutic interventions for individuals at a high genetic risk who are also exposed to severe air pollution levels.

Our finding of a significant additive interaction between genetic susceptibility and air pollution contrasts with a recent report from the UK Biobank by Rhee et al., which observed no significant interaction between PM_2.5_ and genetic risk scores for various CVDs [10]. We note that their endpoint included atrial fibrillation, whereas ours focused on coronary artery disease, heart failure, and stroke. Several methodological differences may explain this discrepancy. First, we specifically tested for and demonstrated interaction on the additive scale using established metrics (RERI, AP, SI), which is considered the relevant scale for assessing biological synergism and public health intervention effects. Second, our analysis included additional key pollutants such as NO_2_ and NO_x_. Another recent study in the UK Biobank by Fu et al. also investigated gene-environment interactions for incident CVD, reporting a synergistic effect between PM_2.5_ and a PRS for coronary artery disease on an additive scale, which aligns with our primary findings and supports the biological plausibility of such interactions [35]. Our positive findings on this front provide stronger evidence for a synergistic biological interplay between an individual's genetic makeup and environmental exposures, suggesting that genetic profiling could indeed identify subgroups that derive disproportionate benefit from air pollution mitigation.

Our PAF estimates translate these relative risks into a potential population health impact. The substantial PAFs, particularly for NO_2_, indicate that a significant fraction of CVD in this population is attributable to air pollution. Crucially, the gradient of increasing PAF across genetic risk strata quantifies the concept of disproportionate burden. This suggests that interventions reducing air pollution exposure would yield the greatest absolute benefit for the sizable subpopulation at high genetic risk, providing a quantitative foundation for genotype-informed public health strategies.

Our findings provide compelling evidence for the significance of air pollution prevention and control, especially for individuals with a high genetic predisposition to CVD. They highlight the persistent adverse effects of air pollution on cardiovascular health, even at levels below WHO guidelines, suggesting that current air quality standards may need revision to better protect public health. Consequently, future endeavors in CVD prevention should prioritize the mitigation of modifiable risk factors, particularly air pollutants. Reinforcing air pollution control measures and promoting personal protective strategies, such as mask usage and the employment of air purifiers, offer a feasible approach to alleviate the burden of CVD. Where feasible, genetic risk assessment tools, including genetic risk detection chips, can be employed to estimate individual genetic susceptibility, facilitating the stratification of genetic risks and the identification of high-risk individuals. For these high-risk individuals, more stringent protective measures may be particularly beneficial, underscoring the importance of personalized prevention strategies. Underpinned by comprehensive air quality improvement, intensifying interventions and management for high-risk individuals holds promise for primary prevention, with significant implications for public health policy and clinical practice.

Our analysis possesses several notable strengths. Firstly, the adoption of a large sample within a prospective study framework to explore the link between air pollutants and incident CVD strengthens the validity of our results. Secondly, employing a Cox proportional hazards model with time-varying exposures mitigates exposure misclassification bias, thereby enhancing the accuracy of our estimates. Thirdly, examining gene-environment interactions offers novel avenues for early CVD prevention and more precise prognostic assessments. However, this study also has certain limitations. First, while we adjusted for major individual and area-level confounders, we were unable to account for certain factors cited in other studies, due to data unavailability or high rates of missingness in the UK Biobank. This potential for residual confounding must be considered. Second, CVD events were defined using ICD codes from administrative records, which, while standard and validated for large epidemiological studies, may be subject to misclassification. Third, the observational nature of our analysis precludes definitive causal inference. Finally, the generalizability of our findings to non-European populations requires further investigation.

## 5 Conclusions

This prospective study provides robust evidence that long-term air pollution exposure increases CVD risk at levels below current WHO guidelines. We further demonstrate a synergistic effect between air pollution and genetic susceptibility, with a disproportionate disease burden borne by individuals at high genetic risk. These findings call for a reevaluation of air quality standards and highlight the potential of integrating genetic information into environmental health protection strategies for targeted risk reduction.

## Ethical approval and consent to participate

The UK Biobank study obtained informed consent from the study participants and approval from its institutional review board. Scientific approval for this study was granted by the UK Biobank (application number:121,022).

## Consent for publication

Not applicable.

## Funding

No funding were received for conducting this study.

## Data availability

The data that support the findings of this study are available from the UK Biobank. Restrictions apply to the availability of these data, which were used under license for the current study, and so are not publicly available.

## CRediT authorship contribution statement

**Yun-Jiu Cheng:** Writing – review & editing, Funding acquisition, Formal analysis, Conceptualization. **Li-Juan Liu:** Formal analysis, Data curation. **Su-Hua Wu:** Investigation, Funding acquisition, Formal analysis. **Li-Chun Wang:** Methodology, Investigation. **Hai Deng:** Project administration, Methodology. **Hui-Qiang Wei:** Resources. **Wei-Dong Lin:** Software, Resources. **Xian-Hong Fang:** Software. **Yi-Jian Liao:** Supervision. **Shu-Lin Wu:** Validation. **Yu-Mei Xue:** Validation. **Yue-Dong Ma:** Writing – review & editing, Visualization. **Yang Wu:** Writing – original draft, Data curation, Conceptualization.

## Declaration of competing interest

The authors declare that they have no known competing financial interests or personal relationships that could have appeared to influence the work reported in this paper.

Yang Wu reports administrative support and article publishing charges were provided by The First Affiliated Hospital of Sun Yat-sen University. Yang Wu reports a relationship with The First Affiliated Hospital of Sun Yat-sen University that includes: employment. If there are other authors, they declare that they have no known competing financial interests or personal relationships that could have appeared to influence the work reported in this paper.

## Appendix Supplementary materials

Image, application 1
