# Supplementary material for: Synergistic effects of genetic susceptibility and air pollution on cardiovascular disease
Source: Am J Prev Cardiol. 2025 Dec 11;25:101381. doi: 10.1016/j.ajpc.2025.101381 (PMC12775991; doi:10.1016/j.ajpc.2025.101381)
Supplement: Supplementary file 1 [file mmc1.docx]

**Synergistic Effects of Genetic Susceptibility and Air Pollution on Cardiovascular Disease**

**Table of Contents**

**Text S1.** Detailed description of polygenic risk score (PRS) construction.

**Table S1.** Summary results for the top 100 SNPs selected by the LASSO model for polygenic risk score calculation.

**Table S2.** Associations between air pollutants concentrations at low levels and the risk of incident CVD among participants in the UK Biobank.

**Table S3.** Associations of PRS with the risk of incident CVD among participants in the UK Biobank.

**Table S4.** Associations between air pollutants and the risk of incident CVD after excluding participants diagnosed with CVD during the first three years of follow-up.

**Table S5.** Associations between air pollutants and the risk of incident CVD in the Fine and Gray competing risk model.

**Table S6.** Associations between air pollutants and the risk of incident CVD in participants without missing covariates.

**Table S7.** Associations between air pollutants and the risk of incident CVD among participants reporting good health at baseline.

**Table S8.** Associations between air pollutant exposure and the risk of incident CVD among participants residing at their current address for > 5 years.

**Table S9.** Associations between air pollutant exposure and the risk of incident CVD in two-pollutant models.

**Table S10.** Stratified analyses of the associations between air pollutant exposure and incident CVD risk by demographic and clinical factors.

**Table S11.** Population Attributable Fraction of CVD Due to Air Pollution Exposure in the Overall Population and by Genetic Risk Stratum.

**Table S12.** Sensitivity analysis: Associations between air pollutants and CVD risk with additional adjustment for area-level deprivation, road proximity, and urban-rural classification

**Fig. S1** Association between PRS and the risk of CVD incidence examined using the Cox proportional hazards regression model.

**Text S1.** Detailed description of polygenic risk score (PRS) construction.

According to the method previously reported in the literature [1], the PRS was calculated as follows:

$$Weighted PRS= \sum_{i=1}^{n} \beta_{i}{SNP}_{i}\times(\frac{N}{Sum(\beta)})$$

Where $\beta$ is per allele log odds ratios for CVD associated with $SNP$, which obtained from the existing GWASs [2]; $SNP$ was documented as 0, 1, and 2 according to the number of risk alleles; $n$ was the total of selected $SNPs$.

The PRS was calculated using genotype data from UK Biobank participants who passed standard quality control (QC). QC included filtering for call rate > 98%, Hardy-Weinberg equilibrium p > 1×10⁻⁶, and sex chromosome aneuploidy checks. The PRS was derived using the bigsnpr R package (version 1.12.2), which implements a penalized logistic regression (LASSO) model on individual-level genetic data, using external GWAS summary statistics as a prior.

The base data for the PRS were the summary statistics from a large-scale genome-wide association meta-analysis for Coronary Artery Disease (CAD) conducted by the CARDIoGRAMplusC4D consortium. We first matched variants present in both the UK Biobank genotype data and the GWAS summary statistics. Linkage disequilibrium (LD) information for the model was estimated from a random subset of 10,000 unrelated UK Biobank participants of European ancestry. The LASSO regression was then run on a separate, non-overlapping set of UK Biobank participants to select informative SNPs and estimate their effect sizes (weights), automatically accounting for LD among variants.

**References**

1. Wang M, Zhou T, Song Y, et al. Joint exposure to various ambient air pollutants and incident heart failure: a prospective analysis in UK Biobank. European heart journal 2021, 42(16):1582-1591.
2. Nikpay M, Goel A, Won HH, et al. A comprehensive 1,000 Genomes-based genome-wide association meta-analysis of coronary artery disease. Nat Genet. 2015;47(10):1121-1130.

**Table S1.** Summary results for the top 100 SNPs selected by the LASSO model for polygenic risk score calculation.

| SNP | CHR | BP | A1 | A2 | Beta | Se | *p* |
| --- | --- | --- | --- | --- | --- | --- | --- |
| rs2891168 | 9 | 22098619 | A | G | -0.193401 | 0.0091877 | 2.29E-98 |
| rs4977574 | 9 | 22098574 | A | G | -0.192934 | 0.0091867 | 6.35E-98 |
| rs10738610 | 9 | 22123766 | A | C | -0.19367 | 0.009292 | 1.78E-96 |
| rs10757275 | 9 | 22106225 | G | A | -0.193037 | 0.0092692 | 2.54E-96 |
| rs10757279 | 9 | 22124630 | A | G | -0.192958 | 0.0093789 | 4.73E-94 |
| rs10757277 | 9 | 22124450 | A | G | -0.192919 | 0.0093811 | 5.69E-94 |
| rs1333048 | 9 | 22125347 | A | C | -0.191445 | 0.0093105 | 5.97E-94 |
| rs10757278 | 9 | 22124477 | A | G | -0.192621 | 0.0093811 | 1.09E-93 |
| rs10757271 | 9 | 22076795 | G | A | 0.180856 | 0.009468 | 2.44E-81 |
| rs10811650 | 9 | 22067593 | A | G | -0.153987 | 0.0092366 | 2.12E-62 |
| rs7028268 | 9 | 22048414 | G | A | -0.137562 | 0.0094474 | 4.98E-48 |
| rs3217992 | 9 | 22003223 | C | T | -0.128317 | 0.009367 | 1.03E-42 |
| rs9349379 | 6 | 12903957 | A | G | -0.131836 | 0.0096527 | 1.81E-42 |
| rs10738604 | 9 | 22025493 | G | A | -0.125862 | 0.0095028 | 4.84E-40 |
| rs1537373 | 9 | 22103341 | G | T | 0.210038 | 0.0159759 | 1.77E-39 |
| rs1333042 | 9 | 22103813 | G | A | 0.209298 | 0.0159553 | 2.60E-39 |
| rs7859362 | 9 | 22105927 | C | T | 0.207873 | 0.0158477 | 2.63E-39 |
| rs1537371 | 9 | 22099568 | A | C | 0.208587 | 0.0159585 | 4.85E-39 |
| rs55730499 | 6 | 161005610 | C | T | -0.316641 | 0.0242403 | 5.39E-39 |
| rs10455872 | 6 | 161010118 | A | G | -0.318598 | 0.0243989 | 5.73E-39 |
| rs10757274 | 9 | 22096055 | A | G | -0.200768 | 0.0155088 | 2.49E-38 |
| rs7859727 | 9 | 22102165 | T | C | 0.206386 | 0.0159607 | 3.01E-38 |
| rs10738607 | 9 | 22088094 | A | G | -0.200924 | 0.0155772 | 4.59E-38 |
| rs10738608 | 9 | 22094796 | C | A | 0.208958 | 0.0163746 | 2.70E-37 |
| rs10757272 | 9 | 22088260 | C | T | -0.200517 | 0.0157402 | 3.58E-37 |
| rs118039278 | 6 | 160985526 | G | A | -0.305337 | 0.0239981 | 4.38E-37 |
| rs1537375 | 9 | 22116071 | C | T | 0.201411 | 0.0159216 | 1.12E-36 |
| rs1412829 | 9 | 22043926 | A | G | 0.122137 | 0.0098384 | 2.19E-35 |
| rs4977757 | 9 | 22094330 | G | A | 0.203806 | 0.0164191 | 2.23E-35 |
| rs10511701 | 9 | 22112599 | C | T | 0.200782 | 0.0162029 | 2.90E-35 |
| rs634537 | 9 | 22032152 | T | G | 0.121111 | 0.009821 | 6.11E-35 |
| rs679038 | 9 | 22029080 | G | A | 0.121085 | 0.00982 | 6.21E-35 |
| rs599452 | 9 | 22027402 | G | A | 0.121046 | 0.0098232 | 6.86E-35 |
| rs613312 | 9 | 22026594 | G | A | 0.121005 | 0.0098265 | 7.60E-35 |
| rs564398 | 9 | 22029547 | T | C | 0.120701 | 0.0098156 | 9.42E-35 |
| rs10811652 | 9 | 22077085 | C | A | 0.194466 | 0.0158749 | 1.68E-34 |
| rs1537370 | 9 | 22084310 | T | C | 0.1903 | 0.0156294 | 4.18E-34 |
| rs9644862 | 9 | 22090936 | G | T | 0.207289 | 0.0170872 | 7.21E-34 |
| rs62560774 | 9 | 22028406 | C | A | 0.125933 | 0.0104554 | 2.07E-33 |
| rs1333036 | 9 | 22043819 | C | T | 0.110307 | 0.0092464 | 8.28E-33 |
| rs10811656 | 9 | 22124472 | C | T | -0.20057 | 0.0168308 | 9.67E-33 |
| rs10115049 | 9 | 22032119 | G | A | 0.109647 | 0.0092149 | 1.2E-32 |
| rs10965215 | 9 | 22029445 | A | G | 0.109519 | 0.0092073 | 1.26E-32 |
| rs6475606 | 9 | 22081850 | T | C | 0.189601 | 0.0159607 | 1.52E-32 |
| rs10116277 | 9 | 22081397 | T | G | 0.189811 | 0.0159813 | 1.56E-32 |
| rs2151280 | 9 | 22034719 | A | G | 0.10975 | 0.0092421 | 1.6E-32 |
| rs4977753 | 9 | 22030027 | T | C | 0.108905 | 0.0092182 | 3.3E-32 |
| rs9644860 | 9 | 22090603 | T | C | 0.20278 | 0.0172175 | 5.1E-32 |
| rs7049105 | 9 | 22028801 | G | A | 0.10822 | 0.0092138 | 7.46E-32 |
| rs10811653 | 9 | 22091069 | T | C | 0.202914 | 0.0173305 | 1.15E-31 |
| rs2210538 | 9 | 22092257 | A | G | 0.20035 | 0.0174054 | 1.16E-30 |
| rs186696265 | 6 | 161111700 | C | T | -0.550351 | 0.0481949 | 3.35E-30 |
| rs581876 | 9 | 22022376 | C | T | 0.10962 | 0.0097298 | 1.92E-29 |
| rs10757265 | 9 | 22048859 | C | T | 0.102875 | 0.0092562 | 1.07E-28 |
| rs9644859 | 9 | 22090521 | G | A | -0.202404 | 0.0182592 | 1.48E-28 |
| rs7874604 | 9 | 22054690 | C | T | 0.109668 | 0.0098971 | 1.55E-28 |
| rs7866503 | 9 | 22091924 | G | T | -0.202877 | 0.0183635 | 2.25E-28 |
| rs7027950 | 9 | 22048391 | T | C | 0.102105 | 0.0092431 | 2.28E-28 |
| rs10757266 | 9 | 22049555 | T | C | 0.10183 | 0.0092431 | 3.17E-28 |
| rs7028570 | 9 | 22048683 | A | G | 0.101738 | 0.0092442 | 3.59E-28 |
| rs1360590 | 9 | 22041443 | C | T | 0.101839 | 0.0092616 | 4E-28 |
| rs615552 | 9 | 22026077 | T | C | 0.107351 | 0.0097798 | 4.94E-28 |
| rs568447 | 9 | 22021615 | A | G | -0.10174 | 0.0092736 | 5.27E-28 |
| rs523096 | 9 | 22019129 | A | G | 0.105282 | 0.0097646 | 4.18E-27 |
| rs944800 | 9 | 22050898 | G | A | 0.112626 | 0.0104533 | 4.55E-27 |
| rs2811713 | 9 | 21999328 | G | A | 0.107374 | 0.0100155 | 8.13E-27 |
| rs10120806 | 9 | 22047945 | C | T | 0.100799 | 0.0094593 | 1.63E-26 |
| rs10811645 | 9 | 22049656 | A | G | 0.1009 | 0.0094756 | 1.77E-26 |
| rs10811643 | 9 | 22024966 | G | A | 0.098462 | 0.0092703 | 2.37E-26 |
| rs1101330 | 9 | 22015465 | C | A | 0.10665 | 0.0100774 | 3.57E-26 |
| rs2106119 | 9 | 22017550 | G | A | 0.097055 | 0.0092605 | 1.06E-25 |
| rs2106120 | 9 | 22017101 | T | G | 0.096727 | 0.0092605 | 1.54E-25 |
| rs3218012 | 9 | 21998660 | A | G | 0.097049 | 0.0092953 | 1.62E-25 |
| rs10811640 | 9 | 22013411 | T | G | 0.096549 | 0.009266 | 2.01E-25 |
| rs573687 | 9 | 22011642 | G | A | 0.105127 | 0.0101056 | 2.41E-25 |
| rs10757263 | 9 | 22013805 | T | C | 0.096081 | 0.0092692 | 3.55E-25 |
| rs496892 | 9 | 22024351 | C | T | 0.096111 | 0.0093127 | 5.69E-25 |
| rs9295128 | 6 | 160751531 | G | T | -0.492954 | 0.048461 | 2.64E-24 |
| rs7528419 | 1 | 109817192 | A | G | 0.11453 | 0.011482 | 1.97E-23 |
| rs643319 | 9 | 22017836 | C | A | 0.092655 | 0.0093061 | 2.37E-23 |
| rs12740374 | 1 | 109817590 | G | T | 0.113549 | 0.011482 | 4.63E-23 |
| rs545226 | 9 | 22012422 | A | G | -0.091856 | 0.009367 | 1.06E-22 |
| rs597816 | 9 | 22021172 | T | C | 0.095514 | 0.0099514 | 8.15E-22 |
| rs9457927 | 6 | 160910282 | A | G | -0.357354 | 0.0373549 | 1.11E-21 |
| rs4714955 | 6 | 12903435 | C | T | 0.098544 | 0.0103935 | 2.51E-21 |
| rs140570886 | 6 | 161013013 | T | C | -0.358095 | 0.0378144 | 2.8E-21 |
| rs1101329 | 9 | 22015997 | C | T | 0.093549 | 0.009947 | 5.22E-21 |
| rs62386818 | 6 | 12923767 | C | A | 0.096274 | 0.0102914 | 8.38E-21 |
| rs62389955 | 6 | 12902248 | A | G | 0.097075 | 0.0103772 | 8.39E-21 |
| rs34343839 | 6 | 12922734 | C | T | 0.095922 | 0.0103066 | 1.32E-20 |
| rs12530250 | 6 | 12911965 | A | G | 0.096448 | 0.0103805 | 1.52E-20 |
| rs7739181 | 6 | 12934687 | G | A | 0.096854 | 0.0104902 | 2.64E-20 |
| rs7454157 | 6 | 12909874 | G | A | 0.090825 | 0.0098634 | 3.31E-20 |
| rs10757264 | 9 | 22019732 | G | A | 0.085077 | 0.0093224 | 7.1E-20 |
| rs490005 | 9 | 22020493 | G | A | 0.084218 | 0.0093192 | 1.61E-19 |
| rs629301 | 1 | 109818306 | T | G | 0.101444 | 0.0114233 | 6.66E-19 |
| rs9369640 | 6 | 12901441 | A | C | 0.089106 | 0.0100535 | 7.78E-19 |
| rs660240 | 1 | 109817838 | C | T | 0.102043 | 0.0115287 | 8.66E-19 |
| rs646776 | 1 | 109818530 | T | C | 0.101049 | 0.0114222 | 9.01E-19 |
| rs7751826 | 6 | 12900977 | T | C | 0.088127 | 0.0100741 | 2.17E-18 |

This table lists the top 100 single-nucleotide polymorphisms (SNPs), ranked by p-value in the base coronary artery disease GWAS (CARDIoGRAMplusC4D), that were selected by the LASSO penalized regression algorithm to construct the polygenic risk score. The full PRS model includes a genome-wide set of SNPs. CHR: chromosome; BP: base position (GRCh37); A1: effect allele in the GWAS; A2: reference allele; Beta: effect size (weight) estimated by the LASSO model; Se: standard error of Beta; p: p-value of the SNP in the base GWAS summary statistics.

**Table S2.** Associations between air pollutants concentrations at low levels and the risk of incident CVD among participants in the UK Biobank.

| Air pollutants | HRs (95% CIs) | *P* values | *P* for trend |
| --- | --- | --- | --- |
| PM_2.5_ |  |  |  |
| PM_2.5_-Q1 | Ref. | -- | **<0.001** |
| PM_2.5_-Q2 | 1.03 (0.99, 1.07) | 0.09 |  |
| PM_2.5_-Q3 | **1.07 (1.03, 1.11)** | **0.001** |  |
| PM_2.5_-Q4 | **1.06 (1.02, 1.10)** | **0.002** |  |
| PM_2.5_, per SD increase | **1.02 (1.01, 1.04)** | **<0.001** | **--** |
| PM_10_ |  |  |  |
| PM_10_-Q1 | Ref. | -- | **<0.001** |
| PM_10_-Q2 | 1.03 (0.99, 1.07) | 0.13 |  |
| PM_10_-Q3 | **1.05 (1.00, 1.09)** | **0.03** |  |
| PM_10_-Q4 | **1.05 (1.01, 1.10)** | **0.02** |  |
| PM_10_, per SD increase | **1.03 (1.01, 1.04)** | **0.002** | **--** |
| NO_2_ |  |  |  |
| NO_2_-Q1 | Ref. | -- | **<0.001** |
| NO_2_-Q2 | **1.05 (1.01, 1.09)** | **0.03** |  |
| NO_2_-Q3 | **1.07 (1.03, 1.11)** | **0.002** |  |
| NO_2_-Q4 | **1.11 (1.06, 1.16)** | **<0.001** |  |
| NO_2_, per SD increase | **1.05 (1.03, 1.06)** | **<0.001** | **--** |
| NO_x_ |  |  |  |
| NO_x_-Q1 | Ref. | -- | **<0.001** |
| NO_x_-Q2 | 1.02 (0.98, 1.06) | 0.39 |  |
| NO_x_-Q3 | **1.06 (1.02, 1.10)** | **0.004** |  |
| NO_x_-Q4 | **1.09 (1.04, 1.13)** | **<0.001** |  |
| NO_x_, per SD increase | **1.04 (1.02, 1.05)** | **<0.001** | **--** |

*P* values, HRs and 95% CIs in bold represent significance at *P* < 0.05.

Cox regression models adjusted for UK Biobank assessment center, age, sex, ethnicity, educational attainment, alcohol consumption status, tobacco consumption status, healthy diet score, physical activity (MET-min/week), body mass index (kg/m^2^), systolic blood pressure (mmHg), and diastolic blood pressure (mmHg), and the presence of hyperlipidemia, hypertension, diabetes, birth year, genotyping batch, and the first ten principal components to account for population heterogeneity.

Abbreviations: HRs, hazard ratios; CIs, confidence intervals; PM_2.5_, particular matter with aerodynamic diameter ≤ 2.5 mm; PM_10_, particular matter with an aerodynamic diameter ≤ 10 mm; NO_2_, nitrogen dioxide; NO_x_, nitrogen oxides; BMI, body mass index.

**Table S3.** Associations of PRS with the risk of incident CVD among participants in the UK Biobank.

| PRS | HRs (95% CIs) | *P* values | *P* for trend |
| --- | --- | --- | --- |
| PRS categories |  |  |  |
| Low genetic risk | Ref. | -- | **<0.001** |
| Intermediate genetic risk | **1.23 (1.20, 1.26)** | **<0.001** |  |
| High genetic risk | **1.56 (1.52, 1.60)** | **<0.001** |  |
| PRS, per SD^a^ increase | **1.23 (1.22, 1.25)** | **<0.001** | -- |

*P* values, HRs and 95% CIs in bold represent significance at *P* < 0.05.

^a^ Standard deviation of PRS was 0.98.

Cox regression models adjusted for UK Biobank assessment center, age, sex, ethnicity, educational attainment, alcohol consumption status, tobacco consumption status, healthy diet score, physical activity (MET-min/week), body mass index (kg/m^2^), systolic blood pressure (mmHg), and diastolic blood pressure (mmHg), and the presence of hyperlipidemia, hypertension, diabetes, birth year, genotyping batch, and the first ten principal components to account for population heterogeneity.

Abbreviations: HRs, hazard ratios; CIs, confidence intervals; PRS, polygenic risk score; PM_2.5_, fine particulate matter with diameter <2.5 μm; PM_10_, particulate matter with diameter <10 μm; NO_2_, nitrogen dioxide; NO_x_, nitrogen oxides; BMI, body mass index; SBP, systolic blood pressure.

**Table S4.** Associations between air pollutants and the risk of incident CVD after excluding participants diagnosed with CVD during the first three years of follow-up.

| Air pollutants | HRs (95% CIs) | *P* values | *P* for trend |
| --- | --- | --- | --- |
| PM_2.5_ |  |  |  |
| PM_2.5_-Q1 | Ref. | -- | **<0.001** |
| PM_2.5_-Q2 | **1.05 (1.02, 1.09)** | **0.001** |  |
| PM_2.5_-Q3 | **1.09 (1.05, 1.12)** | **<0.001** |  |
| PM_2.5_-Q4 | **1.16 (1.12, 1.20)** | **<0.001** |  |
| PM_2.5_, per SD increase | **1.05 (1.04, 1.06)** | **<0.001** | **--** |
| PM_10_ |  |  |  |
| PM_10_-Q1 | Ref. | -- | **<0.001** |
| PM_10_-Q2 | **1.06 (1.03, 1.09)** | **<0.001** |  |
| PM_10_-Q3 | **1.12 (1.09, 1.15)** | **<0.001** |  |
| PM_10_-Q4 | **1.15 (1.11, 1.19)** | **<0.001** |  |
| PM_10_, per SD increase | **1.10 (1.07, 1.12)** | **<0.001** | **--** |
| NO_2_ |  |  |  |
| NO_2_-Q1 | Ref. | -- | **<0.001** |
| NO_2_-Q2 | **1.08 (1.05, 1.11)** | **<0.001** |  |
| NO_2_-Q3 | **1.12 (1.09, 1.15)** | **<0.001** |  |
| NO_2_-Q4 | **1.21 (1.17, 1.25)** | **<0.001** |  |
| NO_2_, per SD increase | **1.69 (1.54, 1.86)** | **<0.001** | **--** |
| NO_x_ |  |  |  |
| NO_x_-Q1 | Ref. | -- | **<0.001** |
| NO_x_-Q2 | **1.09 (1.05, 1.12)** | **<0.001** |  |
| NO_x_-Q3 | **1.09 (1.06, 1.13)** | **<0.001** |  |
| NO_x_-Q4 | **1.17 (1.14, 1.21)** | **<0.001** |  |
| NO_x_, per SD increase | **2.13 (1.83, 2.48)** | **<0.001** | **--** |

*P* values, HRs and 95% CIs in bold represent significance at *P* < 0.05.

Cox regression models adjusted for UK Biobank assessment center, age, sex, ethnicity, educational attainment, alcohol consumption status, tobacco consumption status, healthy diet score, physical activity (MET-min/week), body mass index (kg/m^2^), systolic blood pressure (mmHg), and diastolic blood pressure (mmHg), and the presence of hyperlipidemia, hypertension, and diabetes.

Abbreviations: HRs, hazard ratios; CIs, confidence intervals; PM_2.5_, fine particulate matter with diameter <2.5 μm; PM_10_, particulate matter with diameter <10 μm; NO_2_, nitrogen dioxide; NO_x_, nitrogen oxides; BMI, body mass index; SBP, systolic blood pressure.

**Table S5.** Associations between air pollutants and the risk of incident CVD in the Fine and Gray competing risk model.

| Air pollutants | HRs (95% CIs) | *P*-values | *P* for trend |
| --- | --- | --- | --- |
| PM_2.5_ |  |  |  |
| PM_2.5_-Q1 | Ref. |  | <0.001 |
| PM_2.5_-Q2 | 1.04 (1.01, 1.07) | 0.002 |  |
| PM_2.5_-Q3 | 1.06 (1.03, 1.09) | <0.001 |  |
| PM_2.5_-Q4 | 1.12 (1.09, 1.15) | <0.001 |  |
| PM_2.5_, per SD^a^ increase | 1.04 (1.03, 1.05) | <0.001 |  |
| PM_10_ |  |  |  |
| PM_10_-Q1 | Ref. |  | <0.001 |
| PM_10_-Q2 | 1.05 (1.02, 1.08) | <0.001 |  |
| PM_10_-Q3 | 1.08 (1.05, 1.11) | <0.001 |  |
| PM_10_-Q4 | 1.11 (1.08, 1.14) | <0.001 |  |
| PM_10_, per SD^b^ increase | 1.04 (1.03, 1.06) | <0.001 |  |
| NO_2_ |  |  |  |
| NO_2_-Q1 | Ref. |  | <0.001 |
| NO_2_-Q2 | 1.05 (1.02, 1.08) | <0.001 |  |
| NO_2_-Q3 | 1.08 (1.05, 1.11) | <0.001 |  |
| NO_2_-Q4 | 1.15 (1.12, 1.18) | <0.001 |  |
| NO_2_, per SD^d^ increase | 1.05 (1.04, 1.07) | <0.001 |  |
| NO_x_ |  |  |  |
| NO_x_-Q1 | Ref. |  | <0.001 |
| NO_x_-Q2 | 1.06 (1.03, 1.09) | <0.001 |  |
| NO_x_-Q3 | 1.08 (1.05, 1.11) | <0.001 |  |
| NO_x_-Q4 | 1.13 (1.10, 1.16) | <0.001 |  |
| NO_x_, per SD^e^ increase | 1.03 (1.02, 1.05) | <0.001 |  |

HR, hazard ratio; CI, confidence interval; PM_2.5_, particular matter with aerodynamic diameter ≤ 2.5 mm; PM_10_, particular matter with an aerodynamic diameter ≤ 10 mm; NO_2_, nitrogen dioxide; NO_x_, nitrogen oxides.

Adjusted for the same covariates as the primary Cox model. The competing risk was non-cardiovascular death.

^a^The standard deviation of PM_2.5_ was 1.06 μg/m^3^, ^b^The standard deviation of PM_10_ was 1.92 μg/m^3^, ^c^The standard deviation of NO_2_ was 8.77 μg/m^3^, ^d^The standard deviation of NO*x* was 15.50 μg/m^3^.

**Table S6.** Associations between air pollutants and the risk of incident CVD in participants without missing covariates.

| Air pollutants | HRs (95% CIs) | *P*-values | *P* for trend |
| --- | --- | --- | --- |
| PM_2.5_ |  |  |  |
| PM_2.5_-Q1 | Ref. |  | <0.001 |
| PM_2.5_-Q2 | 1.04 (1.01, 1.07) | 0.01 |  |
| PM_2.5_-Q3 | 1.08 (1.04, 1.11) | <0.001 |  |
| PM_2.5_-Q4 | 1.13 (1.10, 1.17) | <0.001 |  |
| PM_2.5_, per SD^a^ increase | 1.04 (1.03, 1.06) | <0.001 |  |
| PM_10_ |  |  |  |
| PM_10_-Q1 | Ref. |  | <0.001 |
| PM_10_-Q2 | 1.03 (1.00, 1.06) | 0.06 |  |
| PM_10_-Q3 | 1.08 (1.05, 1.11) | <0.001 |  |
| PM_10_-Q4 | 1.06 (1.02, 1.09) | 0.001 |  |
| PM_10_, per SD^b^ increase | 1.02 (1.01, 1.03) | <0.001 |  |
| NO_2_ |  |  |  |
| NO_2_-Q1 | Ref. |  | <0.001 |
| NO_2_-Q2 | 1.05 (1.02, 1.08) | 0.002 |  |
| NO_2_-Q3 | 1.09 (1.05, 1.12) | <0.001 |  |
| NO_2_-Q4 | 1.11 (1.07, 1.14) | <0.001 |  |
| NO_2_, per SD^d^ increase | 1.03 (1.02, 1.04) | <0.001 |  |
| NO_x_ |  |  |  |
| NO_x_-Q1 | Ref. |  | <0.001 |
| NO_x_-Q2 | 1.07 (1.04, 1.10) | <0.001 |  |
| NO_x_-Q3 | 1.09 (1.05, 1.12) | <0.001 |  |
| NO_x_-Q4 | 1.14 (1.11, 1.18) | <0.001 |  |
| NO_x_, per SD^e^ increase | 1.04 (1.03, 1.05) | <0.001 |  |

HR, hazard ratio; CI, confidence interval; PM_2.5_, particular matter with aerodynamic diameter ≤ 2.5 mm; PM_10_, particular matter with an aerodynamic diameter ≤ 10 mm; NO_2_, nitrogen dioxide; NO_x_, nitrogen oxides.

Adjusted for UK Biobank assessment center, age, sex, ethnicity, educational attainment, alcohol consumption status, tobacco consumption status, healthy diet score, physical activity (MET-min/week), body mass index (kg/m^2^), systolic blood pressure (mmHg), and diastolic blood pressure (mmHg), and the presence of hyperlipidemia, hypertension, and diabetes.

^a^The standard deviation of PM_2.5_ was 1.06 μg/m^3^, ^b^The standard deviation of PM_10_ was 1.92 μg/m^3^, ^c^The standard deviation of NO_2_ was 8.77 μg/m^3^, ^d^The standard deviation of NO*x* was 15.50 μg/m^3^.

**Table S7.** Associations between air pollutants and the risk of incident CVD among participants reporting good health at baseline.

|  | Air pollution concentrations (quintiles) | | | | HR (95% CI) for per SD increase | P value for trend |
| --- | --- | --- | --- | --- | --- | --- |
|  | Q1 | Q2 | Q3 | Q4 |  |  |
| PM_2.5_ | 1.00 | 1.06 (1.00-1.13) | 1.09 (1.04-1.14) | 1.13 (1.10-1.06) | 1.06 (1.04-1.07) | <0.001 |
| PM_10_ | 1.00 | 1.05 (1.03-1.07) | 1.09 (1.06-1.13) | 1.13 (1.09-1.17) | 1.05 (1.04-1.08) | <0.001 |
| NO_2_ | 1.00 | 1.05 (1.03-1.07) | 1.08 (1.06-1.11) | 1.07 (1.15-1.21) | 1.05 (1.04-1.07) | <0.001 |
| NO_x_ | 1.00 | 1.06 (1.02-1.09) | 1.08 (1.04-1.10) | 1.13 (1.10-1.17) | 1.03 (1.02-1.05) | <0.001 |

HR, hazard ratio; CI, confidence interval; PM_2.5_, particular matter with aerodynamic diameter ≤ 2.5 mm; PM_10_, particular matter with an aerodynamic diameter ≤ 10 mm; NO_2_, nitrogen dioxide; NO_x_, nitrogen oxides.

Adjusted for UK Biobank assessment center, age, sex, ethnicity, educational attainment, alcohol consumption status, tobacco consumption status, healthy diet score, physical activity (MET-min/week), body mass index (kg/m^2^), systolic blood pressure (mmHg), and diastolic blood pressure (mmHg), and the presence of hyperlipidemia, hypertension, and diabetes.

The standard deviation of PM_2.5_ was 1.06 μg/m^3^, The standard deviation of PM_10_ was 1.92 μg/m^3^, The standard deviation of NO_2_ was 8.77 μg/m^3^, The standard deviation of NO*x* was 15.50 μg/m^3^.

**Table S8.** Associations between air pollutant exposure and the risk of incident CVD among participants residing at their current address for > 5 years.

|  | Air pollution concentrations (quintiles) | | | | HR (95% CI) for per SD increase | P value for trend |
| --- | --- | --- | --- | --- | --- | --- |
|  | Q1 | Q2 | Q3 | Q4 |  |  |
| PM_2.5_ | 1.00 | 1.08 (1.04-1.11) | 1.10 (1.07-1.13) | 1.17 (1.14-1.20) | 1.08 (1.07-1.10) | <0.001 |
| PM_10_ | 1.00 | 1.07 (1.03-1.09) | 1.11 (1.07-1.15) | 1.13 (1.11-1.17) | 1.07 (1.05-1.09) | <0.001 |
| NO_2_ | 1.00 | 1.05 (1.03-1.07) | 1.09 (1.06-1.12) | 1.19 (1.16-1.23) | 1.07 (1.05-1.09) | <0.001 |
| NO_x_ | 1.00 | 1.08 (1.05-1.11) | 1.11 (1.07-1.13) | 1.17 (1.13-1.21) | 1.04 (1.02-1.05) | <0.001 |

HR, hazard ratio; CI, confidence interval; PM_2.5_, particular matter with aerodynamic diameter ≤ 2.5 mm; PM_10_, particular matter with an aerodynamic diameter ≤ 10 mm; NO_2_, nitrogen dioxide; NO_x_, nitrogen oxides.

Adjusted for UK Biobank assessment center, age, sex, ethnicity, educational attainment, alcohol consumption status, tobacco consumption status, healthy diet score, physical activity (MET-min/week), body mass index (kg/m^2^), systolic blood pressure (mmHg), and diastolic blood pressure (mmHg), and the presence of hyperlipidemia, hypertension, and diabetes.

The standard deviation of PM_2.5_ was 1.06 μg/m^3^, The standard deviation of PM_10_ was 1.92 μg/m^3^, The standard deviation of NO_2_ was 8.77 μg/m^3^, The standard deviation of NO*x* was 15.50 μg/m^3^.

**Table S9.** Associations between air pollutant exposure and the risk of incident CVD in two-pollutant models.

|  |  | Air pollution concentrations (quintiles) | | | | |  |  |
| --- | --- | --- | --- | --- | --- | --- | --- | --- |
| Air pollutants | Models | Q1 | Q2 | Q3 | Q4 | HR (95% CI) for per SD increase | | P value for trend |
| PM_2.5_ | +NO_2_ | 1.00 | 1.05 (1.02-1.08) | 1.09 (1.05-1.12) | 1.16 (1.12-1.20) | 1.06 (1.05-1.07) | | <0.001 |
|  | +NO_x_ | 1.00 | 1.04 (1.01-1.07) | 1.06 (1.03-1.10) | 1.12 (1.07-1.16) | 1.04 (1.03-1.06) | | <0.001 |
| PM_10_ | +NO_2_ | 1.00 | 1.03 (1.00-1.06) | 1.06 (1.03-1.09) | 1.02 (0.98-1.06) | 0.99 (0.98-1.01) | | 0.21 |
|  | +NO_x_ | 1.00 | 1.03 (1.00-1.05) | 1.05 (1.02-1.08) | 1.01 (0.98-1.04) | 0.99 (0.98-1.01) | | 0.01 |
| NO_2_ | +PM_2.5_ | 1.00 | 1.03 (1.00-1.06) | 1.05 (1.02-1.09) | 1.06 (1.02-1.10) | 0.99 (0.97-1.00) | | <0.001 |
|  | +PM_10_ | 1.00 | 1.07 (1.04-1.10) | 1.12 (1.09-1.15) | 1.17 (1.13-1.21) | 1.04 (1.02-1.05) | | <0.001 |
| NO_x_ | +PM_2.5_ | 1.00 | 1.05 (1.02-1.08) | 1.06 (1.02-1.09) | 1.09 (1.05-1.14) | 1.01 (0.99-1.02) | | <0.001 |
|  | +PM_10_ | 1.00 | 1.08 (1.05-1.11) | 1.10 (1.07-1.13) | 1.17 (1.13-1.21) | 1.04 (1.03-1.06) | | <0.001 |

HR, hazard ratio; CI, confidence interval; PM_2.5_, particular matter with aerodynamic diameter ≤ 2.5 mm; PM_10_, particular matter with an aerodynamic diameter ≤ 10 mm; NO_2_, nitrogen dioxide; NO_x_, nitrogen oxides.

Adjusted for UK Biobank assessment center, age, sex, ethnicity, educational attainment, alcohol consumption status, tobacco consumption status, healthy diet score, physical activity (MET-min/week), body mass index (kg/m^2^), systolic blood pressure (mmHg), and diastolic blood pressure (mmHg), and the presence of hyperlipidemia, hypertension, and diabetes.

The standard deviation of PM_2.5_ was 1.06 μg/m^3^, The standard deviation of PM_10_ was 1.92 μg/m^3^, The standard deviation of NO_2_ was 8.77 μg/m^3^, The standard deviation of NO*x* was 15.50 μg/m^3^.

**Table S10.** Stratified analyses of the associations between air pollutant exposure and incident CVD risk by demographic and clinical factors.

| Subgroups | PM_2.5_, per SD increase^a^ | | PM_10_, per SD increase^b^ | | NO_2_, per SD increase^c^ | | NO_x_, per SD increase^d^ | |
| --- | --- | --- | --- | --- | --- | --- | --- | --- |
|  | HRs (95% CIs) | *P* for interaction | HRs (95% CIs) | *P* for interaction | HRs (95% CIs) | *P* for interaction | HRs (95% CIs) | *P* for interaction |
| Age |  |  |  |  |  |  |  |  |
| ≥55 years | 1.05 (1.04-1.06) | 0.27 | 1.03 (1.02-1.04) | 0.07 | 1.03 (1.02-1.04) | 0.30 | 1.04 (1.03-1.05) | 0.94 |
| <55 years | 1.05 (1.03-1.07) |  | 1.00 (0.98-1.02) |  | 1.01 (1.00-1.03) |  | 1.03 (1.01-1.05) |  |
| Gender |  |  |  |  |  |  |  |  |
| Male | 1.05 (1.03-1.06) | 0.36 | 1.02 (1.01-1.03) | 0.84 | 1.03 (1.02-1.04) | 0.90 | 1.04 (1.03-1.05) | 0.85 |
| Female | 1.05 (1.04-1.07) |  | 1.02 (1.01-1.04) |  | 1.03 (1.01-1.04) |  | 1.04 (1.02-1.05) |  |
| Ethnicity |  |  |  |  |  |  |  |  |
| White Europeans | 1.05 (1.04-1.06) | **0.01** | 1.03 (1.02-1.04) | **0.003** | 1.03 (1.03-1.04) | **0.001** | 1.04 (1.03-1.05) | **0.003** |
| Non-white Europeans | 0.99 (0.95-1.03) |  | 0.98 (0.94-1.01) |  | 0.98 (0.95-1.01) |  | 0.99 (0.96-1.02) |  |
| Higher education |  |  |  |  |  |  |  |  |
| Yes | 1.03 (1.01-1.05) | **0.001** | 1.00 (0.99-1.02) | **0.003** | 1.00 (0.99-1.02) | **<0.001** | 1.02 (1.00-1.03) | **<0.001** |
| No | 1.06 (1.05-1.07) |  | 1.03 (1.02-1.04) |  | 1.05 (1.04-1.06) |  | 1.05 (1.04-1.06) |  |
| Drinking status |  |  |  |  |  |  |  |  |
| Never | 1.01 (0.97-1.06) | 0.46 | 0.99 (0.95-1.03) | 0.55 | 1.00 (0.96-1.04) | 0.80 | 1.01 (0.97-1.05) | 0.40 |
| Former | 1.03 (0.99-1.07) |  | 1.03 (0.99-1.07) |  | 1.03 (0.99-1.07) |  | 1.06 (0.99-1.13) |  |
| Current | 1.05 (1.04-1.06) |  | 1.02 (1.01-1.03) |  | 1.03 (1.02-1.04) |  | 1.06 (1.04-1.07) |  |
| Smoking status |  |  |  |  |  |  |  |  |
| Never | 1.03 (1.02-1.05) | **<0.001** | 1.01 (1.00-1.03) | **0.02** | 1.02 (1.01-1.04) | **0.04** | 1.03 (1.01-1.04) | **<0.001** |
| Former | 1.06 (1.04-1.07) |  | 1.02 (1.01-1.04) |  | 1.03 (1.01-1.04) |  | 1.04 (1.03-1.05) |  |
| Current | 1.05 (1.03-1.08) |  | 1.03 (1.01-1.06) |  | 1.03 (1.01-1.06) |  | 1.06 (1.03-1.08) |  |
| Healthy diet scores |  |  |  |  |  |  |  |  |
| 0-1 | 1.05 (1.02-1.08) | 0.13 | 1.03 (1.01-1.06) | 0.19 | 1.04 (1.02-1.07) | 0.10 | 1.03 (1.01-1.06) | 0.20 |
| 2-3 | 1.06 (1.04-1.07) |  | 1.03 (1.01-1.04) |  | 1.03 (1.02-1.05) |  | 1.05 (1.04-1.06) |  |
| 4-5 | 1.04 (1.02-1.06) |  | 1.01 (1.00-1.03) |  | 1.02 (1.00-1.03) |  | 1.03 (1.01-1.04) |  |
| Hypertension |  |  |  |  |  |  |  |  |
| Yes | 1.06 (1.04-1.07) | **0.03** | 1.04 (1.03-1.06) | **<0.001** | 1.04 (1.03-1.06) | **0.002** | 1.05 (1.04-1.07) | **0.01** |
| No | 1.04 (1.03-1.06) |  | 1.01 (1.00-1.02) |  | 1.02 (1.01-1.03) |  | 1.03 (1.02-1.04) |  |
| Diabetes |  |  |  |  |  |  |  |  |
| Yes | 1.07 (1.03-1.10) | 0.05 | 1.04 (1.01-1.07) | **0.003** | 1.05 (1.02-1.08) | 0.01 | 1.04 (1.01-1.07) | 0.25 |
| No | 1.05 (1.04-1.06) |  | 1.02 (1.01-1.03) |  | 1.03 (1.02-1.04) |  | 1.04 (1.03-1.05) |  |

HR, hazard ratio; CI, confidence interval; PM_2.5_, particular matter with aerodynamic diameter ≤ 2.5 mm; PM_10_, particular matter with an aerodynamic diameter ≤ 10 mm; NO_2_, nitrogen dioxide; NO_x_, nitrogen oxides.

Adjusted for UK Biobank assessment center, age, sex, ethnicity, educational attainment, alcohol consumption status, tobacco consumption status, healthy diet score, physical activity (MET-min/week), body mass index (kg/m^2^), systolic blood pressure (mmHg), and diastolic blood pressure (mmHg), and the presence of hyperlipidemia, hypertension, and diabetes.

^a^The standard deviation of PM_2.5_ was 1.06 μg/m^3^, ^b^The standard deviation of PM_10_ was 1.92 μg/m^3^, ^c^The standard deviation of NO_2_ was 8.77 μg/m^3^, ^d^The standard deviation of NO*x* was 15.50 μg/m^3^.

**Table S11.** Population Attributable Fraction (PAF) of CVD Due to Air Pollution Exposure in the Overall Population and by Genetic Risk Stratum.

| Air Pollutant | Overall PAF % (95% CI) | Low Genetic Risk PAF % (95% CI) | Intermediate Genetic Risk PAF % (95% CI) | High Genetic Risk PAF % (95% CI) |
| --- | --- | --- | --- | --- |
| PM_2.5_ |  |  |  |  |
| Q2 vs Q1 | 2.1 (1.5, 2.7) | 1.5 (0.8, 2.2) | 2.0 (1.3, 2.7) | 3.5 (2.7, 4.3) |
| Q3 vs Q1 | 3.5 (2.8, 4.2) | 2.2 (1.4, 3.0) | 3.3 (2.5, 4.1) | 5.8 (4.9, 6.7) |
| Q4 vs Q1 | 6.8 (6.0, 7.6) | 4.5 (3.6, 5.4) | 6.5 (5.6, 7.4) | 10.1 (9.1, 11.1) |
| PM_10_ |  |  |  |  |
| Q2 vs Q1 | 2.8 (2.1, 3.5) | 1.9 (1.2, 2.6) | 2.7 (1.9, 3.5) | 4.2 (3.3, 5.1) |
| Q3 vs Q1 | 4.9 (4.1, 5.7) | 3.3 (2.4, 4.2) | 4.7 (3.8, 5.6) | 7.5 (6.5, 8.5) |
| Q4 vs Q1 | 7.2 (6.3, 8.1) | 5.1 (4.1, 6.1) | 6.9 (5.9, 7.9) | 10.8 (9.7, 11.9) |
| NO_2_ |  |  |  |  |
| Q2 vs Q1 | 3.1 (2.4, 3.8) | 2.0 (1.3, 2.7) | 3.0 (2.2, 3.8) | 4.8 (3.9, 5.7) |
| Q3 vs Q1 | 5.4 (4.6, 6.2) | 3.6 (2.7, 4.5) | 5.2 (4.3, 6.1) | 8.3 (7.3, 9.3) |
| Q4 vs Q1 | 9.5 (8.6, 10.4) | 6.5 (5.5, 7.5) | 9.1 (8.1, 10.1) | 14.0 (12.8, 15.2) |
| NO_x_ |  |  |  |  |
| Q2 vs Q1 | 2.5 (1.8, 3.2) | 1.7 (1.0, 2.4) | 2.4 (1.6, 3.2) | 3.9 (3.0, 4.8) |
| Q3 vs Q1 | 4.3 (3.5, 5.1) | 2.9 (2.0, 3.8) | 4.1 (3.2, 5.0) | 6.7 (5.7, 7.7) |
| Q4 vs Q1 | 7.8 (6.9, 8.7) | 5.4 (4.4, 6.4) | 7.5 (6.5, 8.5) | 11.9 (10.8, 13.0) |

HR, hazard ratio; CI, confidence interval; PM_2.5_, particular matter with aerodynamic diameter ≤ 2.5 mm; PM_10_, particular matter with an aerodynamic diameter ≤ 10 mm; NO_2_, nitrogen dioxide; NO_x_, nitrogen oxides.

**Table S12.** Sensitivity analysis: Associations between air pollutants and CVD risk with additional adjustment for area-level deprivation, road proximity, and urban-rural classification

| Air pollutants | HRs (95% CIs) | *P*-values | *P* for trend |
| --- | --- | --- | --- |
| PM_2.5_ |  |  |  |
| PM_2.5_-Q1 | Ref. |  | <0.001 |
| PM_2.5_-Q2 | 1.04 (1.01, 1.07) | 0.005 |  |
| PM_2.5_-Q3 | 1.07 (1.04, 1.10) | <0.001 |  |
| PM_2.5_-Q4 | 1.13 (1.10, 1.17) | <0.001 |  |
| PM_2.5_, per SD^a^ increase | 1.04 (1.03, 1.06) | <0.001 |  |
| PM_10_ |  |  |  |
| PM_10_-Q1 | Ref. |  | <0.001 |
| PM_10_-Q2 | 1.05 (1.02, 1.08) | 0.001 |  |
| PM_10_-Q3 | 1.09 (1.06, 1.12) | <0.001 |  |
| PM_10_-Q4 | 1.13 (1.09, 1.17) | <0.001 |  |
| PM_10_, per SD^b^ increase | 1.05 (1.03, 1.07) | <0.001 |  |
| NO_2_ |  |  |  |
| NO_2_-Q1 | Ref. |  | <0.001 |
| NO_2_-Q2 | 1.05 (1.02, 1.08) | 0.002 |  |
| NO_2_-Q3 | 1.09 (1.06, 1.12) | <0.001 |  |
| NO_2_-Q4 | 1.17 (1.14, 1.21) | <0.001 |  |
| NO_2_, per SD^c^ increase | 1.05 (1.04, 1.07) | <0.001 |  |
| NO_x_ |  |  |  |
| NO_x_-Q1 | Ref. |  | <0.001 |
| NO_x_-Q2 | 1.06 (1.03, 1.09) | <0.001 |  |
| NO_x_-Q3 | 1.08 (1.05, 1.11) | <0.001 |  |
| NO_x_-Q4 | 1.14 (1.11, 1.18) | <0.001 |  |
| NO_x_, per SD^d^ increase | 1.04 (1.02, 1.05) | <0.001 |  |

This model is adjusted for all covariates in the primary model (UK Biobank assessment center, age, sex, ethnicity, educational attainment, alcohol consumption, smoking, healthy diet score, physical activity, BMI, blood pressure, hyperlipidemia, hypertension, and diabetes) plus the Townsend deprivation index, proximity to a major road (≤200 m vs. >200 m), and urban-rural classification.

Abbreviations: HR, hazard ratio; CI, confidence interval; SD, standard deviation (PM2.5: 1.06 μg/m³; PM10: 1.92 μg/m³; NO2: 8.77 μg/m³; NOx: 15.50 μg/m³).


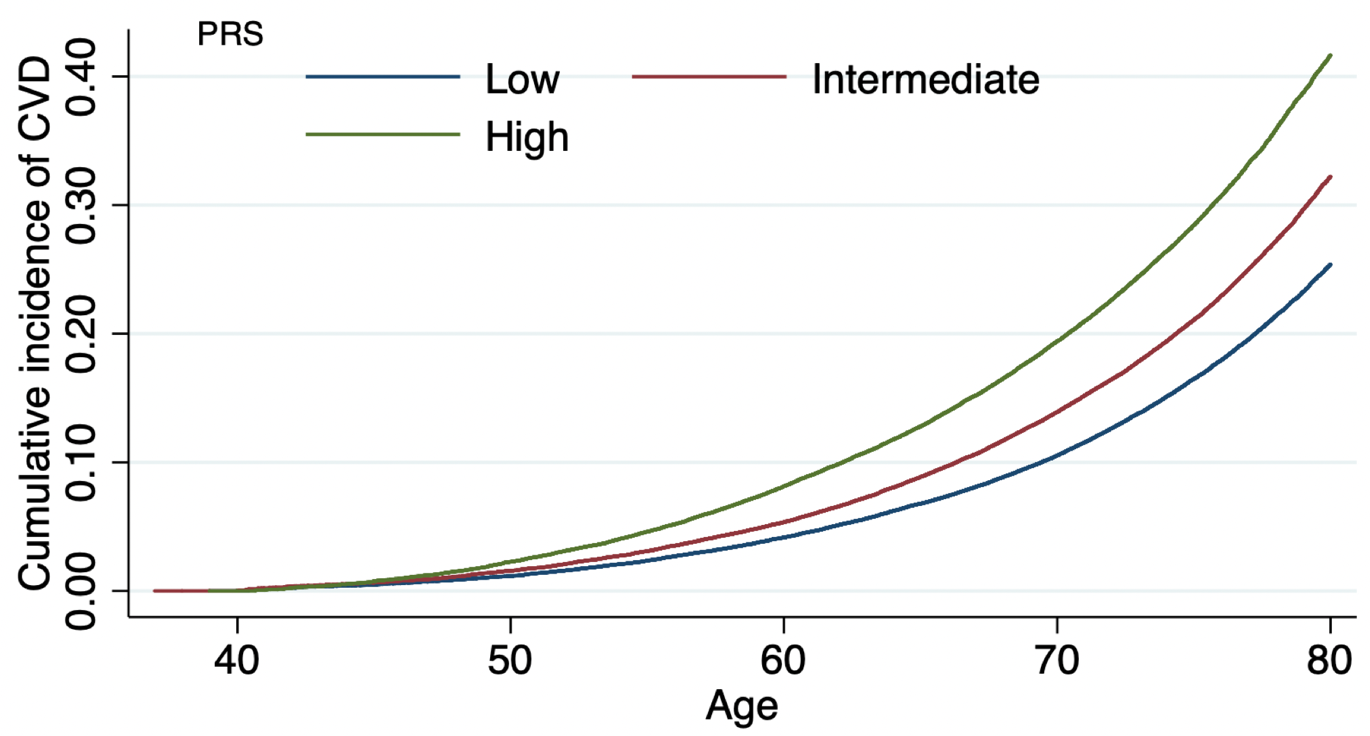


**Fig. S1** Association between PRS and the risk of CVD incidence examined using the Cox proportional hazards regression model. Abbreviations: CVD, cardiovascular disease; PRS, polygenic risk score.
